# Toxicity and Biodistribution of the Serotype 2 Recombinant Adeno-Associated Viral Vector, Encoding Aquaporin-1, after Retroductal Delivery to a Single Mouse Parotid Gland

**DOI:** 10.1371/journal.pone.0092832

**Published:** 2014-03-25

**Authors:** Dariya Momot, Changyu Zheng, Hongen Yin, Reem H. Elbekai, Molly Vallant, John A. Chiorini

**Affiliations:** 1 Molecular Physiology and Therapeutics Branch, National Institute of Dental and Craniofacial Research, National Institutes of Health, Bethesda, Maryland, United States of America; 2 Toxicology Division, BioReliance by SAFC, Rockville, Maryland, United States of America; 3 National Toxicology Program, National Institute of Environmental Health Sciences, National Institutes of Health, Research Triangle Park, North Carolina, United States of America; Justus-Liebig-University Giessen, Germany

## Abstract

In preparation for testing the safety of using serotype 2 recombinant adeno-associated vector, encoding Aquaporin-1 to treat radiation-induced salivary gland damage in a phase 1 clinical trial, we conducted a 13 week GLP biodistribution and toxicology study using Balb/c mice. To best assess the safety of rAAV2hAQP1 as well as resemble clinical delivery, vector (10^8^, 10^9^, 10^10^, or 4.4×10^10^ vector particles/gland) or saline was delivered to the right parotid gland of mice via retroductal cannulation. Very mild surgically induced inflammation was caused by this procedure, seen in 3.6% of animals for the right parotid gland, and 5.3% for the left parotid gland. Long term distribution of vector appeared to be localized to the site of cannulation as well as the right and left draining submandibular lymph nodes at levels >50 copies/μg in some animals. As expected, there was a dose-related increase in neutralizing antibodies produced by day 29. Overall, animals appeared to thrive, with no differences in mean body weight, food or water consumption between groups. There were no significant adverse effects due to treatment noted by clinical chemistry and pathology evaluations. Hematology assessment of serum demonstrated very limited changes to the white blood cell, segmented neutrophils, and hematocrit levels and were concluded to not be vector-associated. Indicators for liver, kidney, cardiac functions and general tissue damage showed no changes due to treatment. All of these indicators suggest the treatment is clinically safe.

## Introduction

Radiation therapy is often used for head and neck cancer treatment, however, it is also known to cause irreversible damage of the salivary glands. Radiation-induced salivary hypofunction may lead to xerostomia, dysphasia, discomfort, and oral infections, all of which can significantly reduce patients’ quality of life [1]. It further impairs oral tissue repair, upper gastrointestinal tract protection, and protein production [2]. There currently is no effective treatment available to reverse this damage.

The human aquaporin-1 gene (Human AQP1 or hAQP1) was the first water channel protein to be characterized and is considered to be the archetypal molecular water channel. Human AQP1 is a 28-kDa membrane monomeric protein that exists in cell membranes as a homotetramer. Each individual monomer can function as a water channel, i.e., facilitate the extremely rapid movement of water in response to an osmotic gradient. Human AQP1 is constitutively “open” and can move water in either direction as soon as an osmotic gradient is imposed, depending on the gradient. Additionally, hAQP1 is widely distributed in a variety of tissues, including red blood cells, renal proximal tubules, choroid plexus, non-pigmented epithelium of the eye, cholangiocytes, and capillary endothelium in numerous organs. In several tissues it is extremely abundant; e.g., every human red blood cell contains ∼150,000 copies of monomeric AQP1. Indeed, AQP1 represents ∼2.4% of total membrane protein in red blood cells, compared with ∼1% in the kidney cortex.

A promising relief for irradiation side effects is the use of gene therapy for tissue repair or engineering. A clinical trial using adenoviral vector encoding human aquaporin-1 (hAQP1) was recently completed and resulted in an increase in saliva flow in 5 out of 11 patients [3]. However, the transgene expression of adenoviral vector in the salivary glands is relatively short lived, lasting <1 month in rats [4]. Extending expression in the salivary gland is reported with adeno-associated virus (AAV) vectors. Thus, the clinical and biological effects of rAAV2hAQP1 delivery to the mouse parotid gland were evaluated and compared to the previously tested AdhAQP1.

AAV vector was administered to mouse parotid gland, and clinical and pathological evaluations performed over the 13-week period following GLP practices and procedures. Careful analysis included clinical chemistry, hematology, gross and microscopic pathology evaluations, analysis of neutralizing antibody formation, and tissue distribution of the vector. The results and conclusions of the study are presented in this paper.

## Materials and Methods

**Figure 1:**
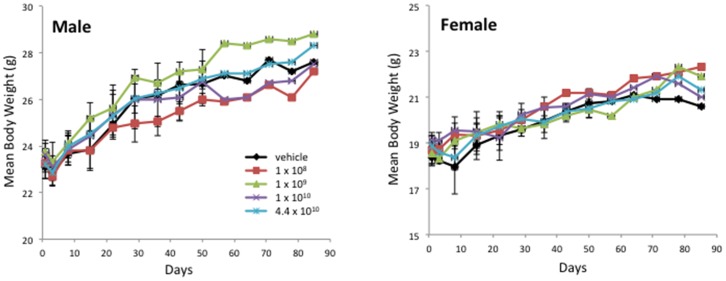
Mean body weight per group for male (left) and female (right) Balb/c mice over the 13 wk rAAV2hAQP1 toxicology study. Vector concentrations are listed as particles/gland. A significant difference in body weight between the vehicle and vector treated groups was observed on day 3 for Cohort A males treated with 1×10^9^ vp/gland (108% of control value) (p<0.05).

**Figure 2:**
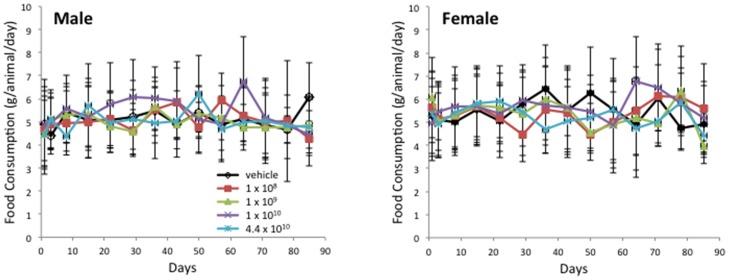
Mean food consumption per group for male (left) and female (right) Balb/c mice over the 13 wk rAAV2hAQP1 toxicology study. Vector concentrations are listed as particles/gland. A significant difference in food consumption was observed in Cohort B females treated with the highest dose, 4.4×10^10^ vp/gland, compared to the vehicle treated mice on week 5 (153% of control value) (n = 5) (p<0.05).

**Figure 3:**
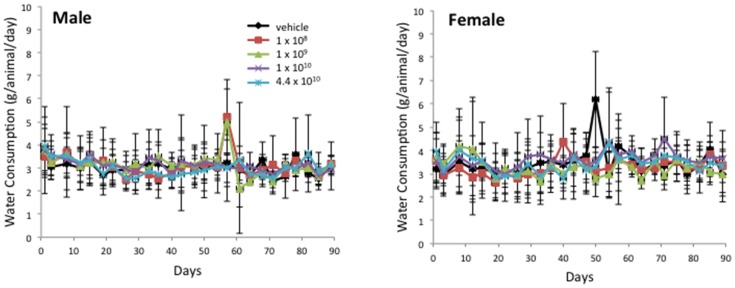
Mean water consumption per group for male (left) and female (right) Balb/c mice over the 13 wk rAAV2hAQP1 toxicology study. Vector concentrations are listed as particles/gland.

All Balb/c mice for this GLP rAAV2hAQP1 toxicity and biodistribution study were obtained from Taconic Farms, Germantown, NY. Animals were 7 weeks of age upon arrival and were quarantined for 28–31 days (males) and 35–38 days (females). They were weighed individually within 48 hours of arrival, on days 1, 3, 8, and weekly thereafter during 92-day study period. Food consumption was determined for days 1–3, 3–8, and weekly thereafter. Water consumption was determined on the day of sacrifice (days 3, 29, 57, and 92) for animals scheduled to be sacrificed and on days 1–3, 3–8, and twice weekly (Mondays and Thursdays) thereafter for the remaining population. Saliva was collected on days 3, 29, 57, and 92 after blood collection. All animals were individually observed for mortality and moribundity twice daily, in the morning before 10:00 am and in the afternoon after 2:00 pm, at least 6 hrs apart, including holidays and weekends. Formal clinical observations were performed weekly and at the time of animal removal. The animals were separated into vehicle and 4 treatment groups. There were four cohorts, corresponding to the day of the sacrifice: Cohort A (day 3), Cohort B (day 29), Cohort C (57), Cohort D (92). Mean values per treatment groups shown in Fig. 1–3 for mean body weight (MBW), mean food consumption (MFC), and mean water consumption (MWC), respectively, were determined based on mean values for that day per cohort per group per gender (n = 5, Cohort A-C; n = 6, Cohort D) for males and females. Thus, n = 105 for days 1–3, n = 80 for days 8–22, n = 55 for days 29–50, n = 30 for days 59–92.

### Ethics statement

The study was conducted in accordance with BioReliance’s Quality Assurance Program following the U.S. EPA and U.S. FDA Good Laboratory Practice Regulations. It also followed the Specifications for the Conduct of Studies to Evaluate the Toxicologic and Carcinogenic Potential of Chemical, Biological, and Physical Agents in Laboratory Animals for the National Toxicology Program (NTP). The study was conducted in an AAALAC accredited facility under the Institutional Animal Care and Use Committee Protocol approval #325. The use of Balb/c mice was selected because no non-mammalian test or in vitro method to the current knowledge can provide the same degree of specificity and reliability as the mammalian model.

### rAAV2hAQP1 vector

The vector concentrations were determined based on the previous study of AAV2 in miniature pigs (minipigs) [5]. AAV2 vector was tested in minipigs at concentrations of 10^10^, 10^11^, and 3×10^11^ vector particle/gland (vp/gland) in a total volume of 4 ml. Peak transduction was found at 1×10^11^ vector particle/gland or a concentration of 7.5×10^7^ vp/μl.

Considering the relative difference in parotid gland fill volume for minipig and mouse (4 ml vs 20 μl), the highest administered dose for the mouse parotid gland was 4.4×10^10^ vp/gland or 2.2×10^9^ vp/μl. Stock concentrations of rAAV2hAQP1 vector were diluted with saline 0.9% Sodium Chloride for Injection, USP (Baxter, NC) to obtain the final dose of 1×10^8^, 1×10^9^, 1×10^10^, 4.4×10^10^ vp/gland.

### Treatment

A total of 125 animals were randomly distributed to one of the 7 groups (Table 1) by their body weight using a computer generated randomization program, Provantis. There were 21 animals per group per gender for groups 1–5, and 10 animals per group per gender for groups 6–7. Treatment was staggered for each sex by one week, with animals from each group staggered consecutively over 4 days. For groups 6 and 7, all 10 animals per group were started on the same day.

**Table 1:** Treatment groups for AdhAQP1 toxicology and biodistribution study.

Group number		Total no. of animals	
	Treatment	Male	Female
1	Vehicle (Saline)	21	21
2	rAAV2hAQP1, 1×10^8^	21	21
3	rAAV2hAQP1, 1×10^9^	21	21
4	rAAV2hAQP1, 1×10^10^	21	21
5	rAAV2hAQP1, 4.4×10^10^	21	21
6	Vehicle (Saline)	10	10
7	rAAV2hAQP1, 4.4×10^10^	10	10

Every animal was anesthetized by intramuscular injection with 5 mL/kg of cocktail containing ketamine 43 mg/kg, xylazine 8.5 mg/kg, and acepromazine 1.4 mg/kg, using additional anesthetic as needed. A PE 10 polyethylene cannula was inserted into the right parotid duct while holding the oral cavity open with two suture hooks. After cannulation, each animal was given 0.5 mg/kg atropine (Sigma-Aldrich, St. Louis, MO) intramuscularly, while the cannula was glued in a fixed position. After 10 minutes, appropriate dose of rAAV2hAQP1 vector or saline was administered into the cannula with an insulin syringe. The cannula was removed 10 minutes after the vector or saline injection. A different cannula and syringe were used for every animal. Saline was also added to the eyes to prevent dryness.

### Collection of saliva, blood, salivary glands, and other tissues

Blood for clinical pathology (hematology and clinical chemistry) evaluations, anti-rAAV2hAQP1 vector antibody detection, and Q-PCR analysis was obtained from the retro-orbital sinus of animals anesthetized with 70% CO_2_/30% O_2_. Animals were bled in the morning of the scheduled necropsy date on days 3, 29, 57, and 92. Of the 600 μL of blood collected from each mouse per group per day into K2EDTA tubes, 150 μL were submitted for clinical pathology evaluation, 100 μL for Q-PCR analysis of AAV2hAQP1, and remainder for antibody analysis. Animals were given 0.5 mL of supplemental water by gavage prior to being returned to their designated cages. Hematology parameters included erythrocyte count, red blood cell distribution width (RDW), mean corpuscular volume, erythrocyte morphologic assessment, hemoglobin, leukocyte count, hematocrit, leukocyte differential, reticulocyte count (%), mean corpuscular hemoglobin (MCH), mean corpuscular hemoglobin concentration (MCHC), platelet count, and mean platelet volume.

Whole blood from groups 6 (vehicle) and 7 (4.4×10^10^ vp/gland) on days 3 and 57 was submitted to clinical pathology for supplemental evaluation of alanine aminotransferase (ALT), blood urea nitrogen (BUN), lactate dehydrogenase (LDH), creatine phosphokinase (CPK), albumin, alkaline phosphatase, cholesterol, creatine kinase, creatinine, glucose, serum amylase, sorbitol dehydrogenase, total protein, triglycerides, total bile acids, sodium and chloride.

Saliva samples were collected 4 hrs after whole blood collection from five animals per groups 1–5 on days 3 (48 hr after vector administration), 29 and 57, and 6 animals per group on day 92. All animals were allowed to consume food and water freely prior to the scheduled procedure. After being anesthesized with a cocktail of ketamine, xylazine and acepromazine, all animals were subcutaneously administered 1 mL/kg freshly prepared pilocaprine (0.5 mg/kg) in the nape of the neck. Additional pilocarpine was used as needed. Saliva was collected over 20 min by drawing it through capillary tubes from the oral cavity into chilled 1.2 mL microcentrifuge tubes and stored at ≤–20°C. 20–50 μL saliva samples were submitted for vector distribution analysis with Q-PCR (BioReliance, MD).

To assess if the vector distributed past the site of injection, the following tissues were also provided for Q-PCR analysis: left and right draining submandibular lymph nodes, left and right submandibular salivary glands, brain, ovaries, heart, liver, left and right parotid salivary gland, lung, duodenum, tongue, spleen, testes, kidney. Tissues were snap frozen in liquid nitrogen and stored below –20°C.

Tissues for histopathological examination were also collected and fixed in 10% neutral buffered formalin on Days 3, 29, 57, and 92. Tissues embedded, sectioned, stained with hematoxylin and eosin, and evaluated microscopically.

### Distribution of rAAV2hAQP1 in tissue, blood and saliva

To determine the distribution of rAAV2hAQP1 vector in animal blood, saliva, and tissue, TaqMan technology was used to amplify the original DNA fragment template *in vitro*. All samples were stored below –20°C until DNA extraction. Blood and saliva DNA was isolated using QIAamp Blood Kit (Qiagen) and measured by volume. Tissue samples were analyzed separately. Whole tissues, up to 25 mg (± 5 mg) by weight, were excised and lysed with the Proteinase K and lysis buffer solution for at least 3 hrs at 56°C ± 2°C. After the incubation period, samples were homogenized using Qiagen Tissuelyzer and DNA was extracted from 200 μL of the lysate using QIAsymphony DNA Mini Kit (Qiagen). Tissue DNA was eluted to 100 μL. DNA concentration for all samples was quantified at 260 nm absorbance wavelength using ABI PRISM 7900HT instrument and data analyzed by the 7900HT Sequence Detection software (Applied Biosystems, Foster City, CA).

To determine rAAV2hAQP1 vector presence, a plasmid was used to create a standard curve as well as spike the samples for PCR validation. A master stock was prepared at 1×10^10^ copies/μL, and serially diluted into seven concentrations ranging from 5×10^6^ to 5×10^0^ copies/μL. Q-PCR assay was optimized to detect 50 copies of rAAV2hAQP1 DNA for the given concentrations and primers. The primer sequences are listed below.

Forward primer, 90311F2: 5′-CGC GAA TTC GAG CTC GGT-3′

Reverse primer, 90311R2: 5′-GCT GCC GGG TGC TCAA-3′

Probe, 90311 FAM-MGB2: 5′-CCA GCT CTC AGA GGGA-3′

PCR amplification was performed on up to 1.0 μg for tissue samples, and 10 μL for collected saliva and/or blood. Vector was quantified in three separate reactions performed for each test sample DNA, of which one reaction was spiked with 500 copies of rAAV2hAQP1 DNA to serve as the spiked test sample control. The sample result was considered informative when the respective spiked test sample control produced a fluorescent signal within the quantifiable range of the standard curve. All spiked test samples produced expected amplification signals and any observed matrix interference was relieved by testing a lower dilution of the test sample DNA. All positive control standard point replicates were tested three times as well. The C_T_ value of the no template control was determined to be 40. A total of 16 tissues were tested: left and right draining submandibular lymph nodes, left and right submandibular salivary glands, brain, ovaries, heart, liver, left and right parotid salivary gland, lung, duodenum, tongue, spleen, testes, kidney.

### Determination of neutralizing antibody titers in serum

To determine the presence of neutralizing antibodies (NAbs) as a host immune response to the rAAV2hAQP1 administration, serum from mice bled at day 3 and day 29 per dose was assayed. 2×10^5^ Cos cells were plated in 96-well plates and cultured for 24 hr. The following day, DMEM medium containing 2×10^7^ particles AAV2-luc (100 particles/cell) was prepared. 50 μL of mouse serum at dilutions from 1∶4 to 1∶2048 was added to the DMEM medium and incubated for 30 min at 37°C. The mixture was added to the cells and incubated for 1 hr at 37°C. Following another 24 hr incubation, cells were lysed with 50 μl of lysis buffer (Promega, Madison, WI). The lysate was then added to 100 ml of luciferase substrate and the luciferase activity was measured with a luminometer in duplicate.

## Results

### General indicators of animal health

There were no adverse effects associated with rAAV2hAQP1 vector treatment in Balb/c mice over the 13-week study period. All animals appeared to be thriving throughout the trial period, as suggested by the weight gain, food consumption, and water consumption, with no gender nor vector-treatment associated differences. All female mice survived until the scheduled sacrifice on day 3, 29, 57, or 92. A total of two male mice out of 125 died unexpectedly, one on day 23, from group 4, and one on day 26, from group 5. The deaths are not believed to be due to the vector treatment.

The mean body weight increased for males and females with little difference between the treatment groups, suggesting healthy animal growth regardless of the vector concentration (Fig. 1). The only exception is on day 3, group 3 body weight was significantly increased for males, to 108%, compared to the vehicle group. Food consumption remained consistent throughout the study (Fig. 2). All individual food consumption values above 8.5 g/animal/day were excluded. A statistically significant increase in food consumption (153%) was only seen between group 5 and the vehicle group females on day 29. No significant changes in water consumption were observed, suggesting treatment with rAAV2hAQP1 had no effect on either food or water consumption in Balb/c mice (Fig. 3). Minimal clinical signs were seen in only a few animals but none of these signs are believed to be related to vector treatment due to low number of animals affected and/or lack of dose response.

### Histopathologic evaluation

A total of 15 tissues were examined microscopically in test animals. On day 3, minimal to mild inflammation of the right parotid gland was present in 3 male mice, and of the left parotid gland in 6 male mice from all dose groups (Table 2). In female mice, minimal to mild inflammation of the right parotid gland was found in the total of 6 mice and of the left parotid gland in 7 mice. Inflammation of the right and left parotid glands were seen in equal frequencies and intensities across all groups. At day 92, only one male mouse, from group 2, had inflammation, which was limited to the right parotid gland. Left and right parotid gland inflammation was similar and mainly did not proceed past day 3. This suggests that inflammation of the right and left parotid glands was likely to be induced by surgical suture needles used during the procedure to hold open the mouse oral cavity and not by treatment. Cytoplasmic vacuolization of the epithelial cells was also present in the parotid gland concomitantly with inflammation. In instances where cytoplasmic vacuolization was not accompanied by inflammation, it was diagnosed separately (Table 2). There was a trend in the increase in incidence of vacuolization with time, across all groups, although there was no difference in the incidence or severity of cytoplasmic vacuolization in the vehicle and test article treated groups. Cytoplasmic vacuolization is likely a result of the inflammatory process, and hence secondary to the surgical procedure. The surgical suture needles will not be necessary in humans. No lesions in any other tissue examined were considered treatment related.

**Table 2:** Summary of microscopic changes observed in the parotid gland.

	Males					Females				
	Grp 1	Grp 2	Grp 3	Grp 4	Grp 5	Grp 1	Grp 2	Grp 3	Grp 4	Grp 5
*Day 3*										
Left Parotid Gland										
Inflammation	1	2	-	2	1	2	-	1	2	2
Vacuolization	-	-	-	-	1	1	-	-	-	-
Right Parotid Gland										
Inflammation	1	-	-	1	1	1	3	1	-	1
Vacuolization	-	-	-	-	-	2	-	-	-	-
*Day 29*										
Left Parotid Gland										
Inflammation	-	-	-	-	-	-	-	-	-	-
Vacuolization	-	3	2	1	2	-	-	-	-	1
Right Parotid Gland										
Inflammation	-	-	-	-	-	-	-	-	-	-
Vacuolization	-	2	2	1	2	-	-	-	-	-
*Day 57*										
Left Parotid Gland										
Inflammation	-	-	-	-	-	-	-	-	-	-
Vacuolization	-	2	1	1	1	-	1	-	1	-
Right Parotid Gland										
Inflammation	-	-	-	-	-	-	-	-	-	-
Vacuolization	-	2	-	1	2	-	1	-	1	-
*Day 92*										
Left Parotid Gland										
Inflammation	-	1	-	-	-	-	-	-	-	-
Vacuolization	2	-	2	2	2	-	2	4	-	-
Right Parotid Gland										
Inflammation	1	1	-	-	-	-	-	-	-	-
Vacuolization	3	1	1	2	1	-	1	2	-	-

Summary of microscopic changes observed in the parotid gland. Data shown represent the number of animals with microscopic evidence of inflammation or cytoplasmic vacuolization. Microscopic changes observed in the parotid gland.

### Hematology and clinical chemistry

**Figure 4:**
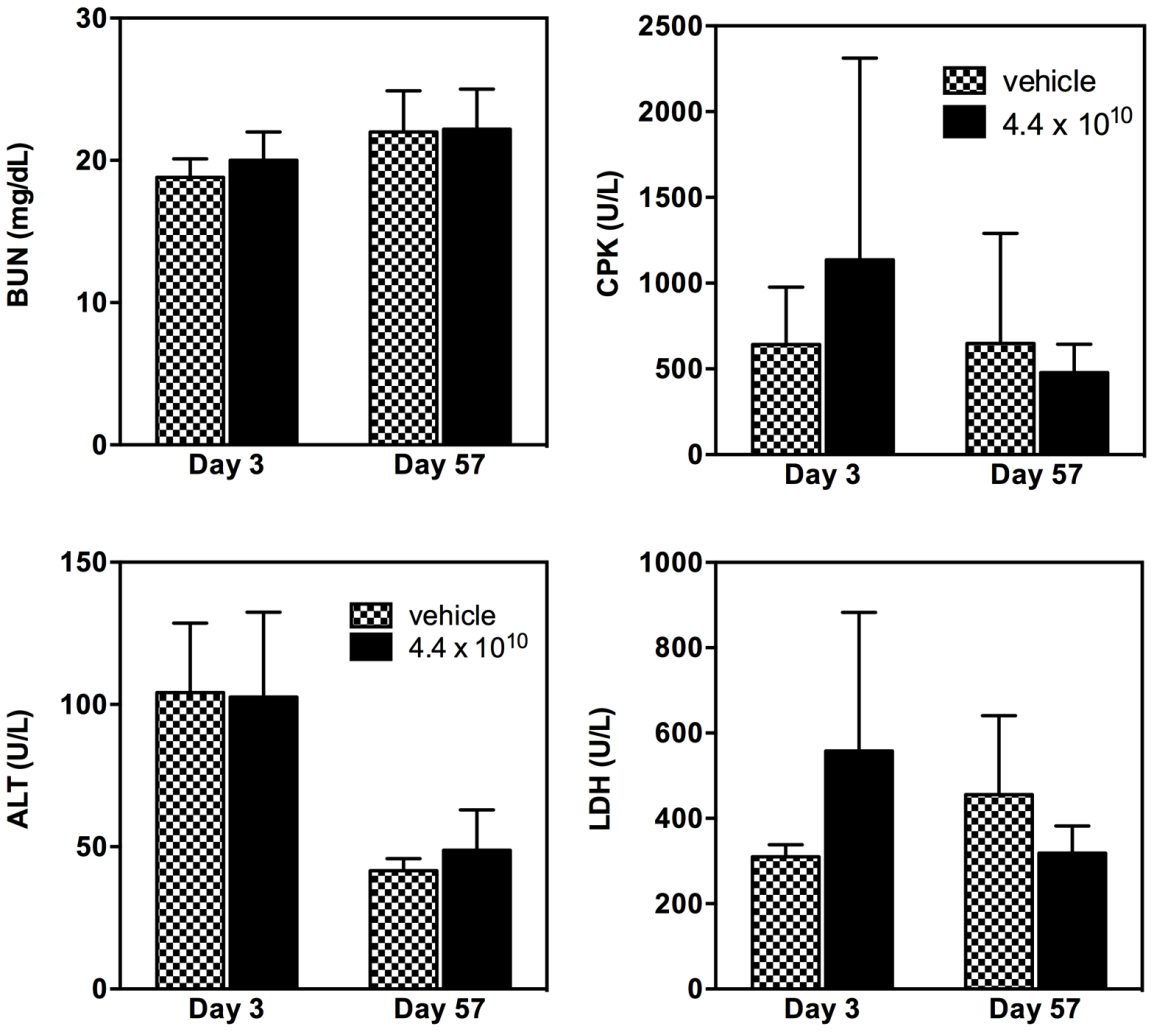
Clinical chemistry analysis of organ function in male Balb/c mice treated with vehicle or 4.4×10^10^ vp/gland of rAAV2hAQP1. Tissue damage indicators presented are blood urea nitrogen (BUN) for kidney damage, creatine phosphokinase (CPK) for heart damage, alanine aminotransferase (ALT) for liver damage, and lactate dehydrogenase (LDH) for general tissue damage. Data are based on mean values ± SD. Vector concentrations are listed as particles of rAAV2hAQP1/gland.

Clinical chemistry and hematology evaluations detected no significant vector-induced differences in health between the vehicle and treated groups (Fig. 4). Recombinant AAV2hAQP1 vector had no effect on the function of heart, as measured by the creatine phosphokinase (CPK) levels; liver, alanine aminotransferase (ALT) levels; and kidney, blood urea nitrogen (BUN) levels. No significant biologic differences were noted between males and females (data not shown). Lack of clinical changes were further confirmed with no statistically significant difference noted between treatment groups and/or genders in lactate dehydrogenase (LDH) levels used to measure general tissue damage.

**Figure 5:**
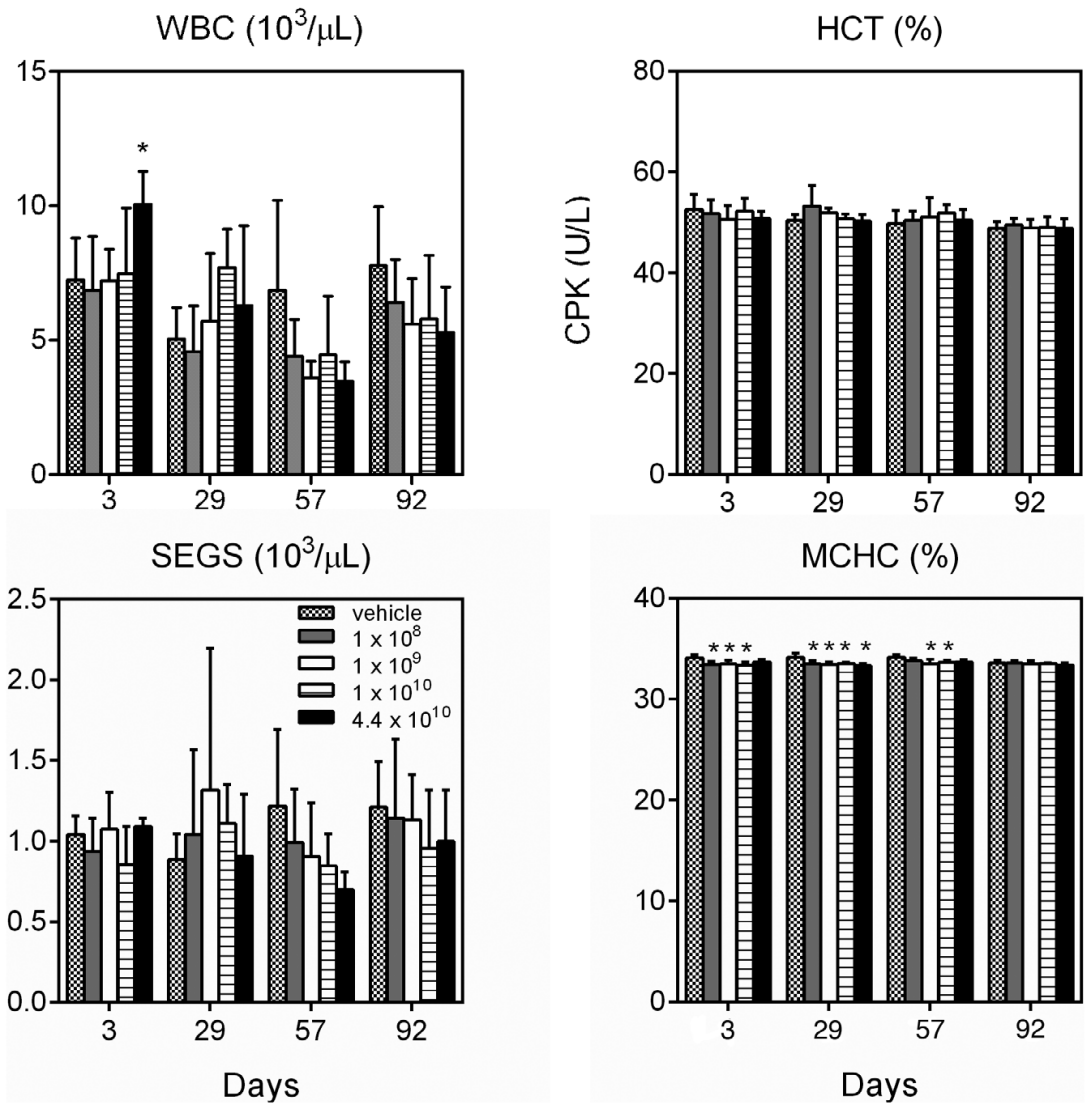
Hematology analysis for male Balb/c mice treated with vehicle or listed doses of rAAV2hAQP1/gland. Evaluation of white blood cells (WBC), hematocrit (HCT), and segmented neutrophils (SEGS) levels had no strong dose or time dependent response in either males or females. WBC count was significantly elevated in the group treated with 4.4×10^10^ vp/gland compared with the control on day 3 in males (p<0.05). Mean Corpuscular HGB Concentration (MCHC), however, was significantly lower in vector treated animals from some groups compared to the vehicle group after the treatment (days 3, 29, and 57). Females had similar results, with decreased MCHC seen in groups 3, 5 on day 29; and group 2, 3–4 on day 92 only (data not shown).

Minor hematological differences in HCT (hematocrit), WBC (white blood cell), and SEGS (segmented neutrophils) are illustrated by Figure 5, but were concluded to be random and not vector dose or gender related. Group 5, receiving the highest vector dose, had significantly higher WBC concentration than the control group 1 on day 3 in males (p<0.05). On day 57, the WBC count in females of group 3 was significantly lower than the control for the same day, and compared to the count from the same group on day 3. SEGS levels were also slightly declined in the highest dose treated group compared to the control on day 3 in females.

WBC levels were significantly lower on day 57 than on day 3 for group 3 males, but not females. WBC levels were also significantly elevated on day 3 compared with day 57 or day 92 for group 5 males. SEGS levels in males did not significantly differ with time or treatment, except for group 5 on day 57, when SEGS(10^3^/μl) was significantly lower than on day 3.

A statistically significant change was observed in the mean corpuscular HGB concentration (MCHC) levels. Groups 3 and 4 males had decreased levels of MCHC on days 3, 29, 57; group 2 on days 3 and 29; and group 5 on day 29 only. Females (data not shown), had decreased MCHC in groups 3 and 5 on day 29; and in groups 2, 3, 4 on day 92 only. Overall these changes were small (1.2 to 2.4%) and were not detected in all vector treated groups.

There were no major differences in the hematology report between males and females. Female mice had significantly higher WBC values for day 57 group 5, HCT values for day 92 group 1, SEGS values day 3 group 1, and SEGS values day 57 group 5 compared to males. Because the changes in hematology evaluations were limited, they were concluded to be random and not vector induced.

### Neutralizing Antibodies after rAAV2hAQP1 administration

Intramuscular and intranasal routes of rAAV2 vector delivery were previously shown to initiate a mild host immune response. Because salivary glands are part of the mucosal immune system, it was expected for the host to develop an immune response to the rAAV2hAQP1 vector in the form of neutralizing antibodies (NABs) [6].

A mixture of mouse serum from 5 animals per gender group was used for measurements, thus an average value per group was determined. There were no neutralizing antibodies (NAB) detected on day 3 in either male or female mice, regardless of the treatment. The control group had no detectable levels of NAB on day 29, while groups with the rAAV2 vector had a dose-dependent increase in NAB levels (Table 3).

**Table 3:** Number of animals in each gender group (n =  5 for males, n = 5 for females, for a total of 10 animals per dose group per day 3 and day 29) that exhibited immune response to the AAV by producing neutralizing antibodies (NAB) against the AAV capsid.

Group	Male	Female
1	0	0
2	1∶4	1∶8
3	1∶8	1∶16
4	1∶1024	1∶1024
5	1∶2048	1∶2048

There were no detectable NAB on Day 3 and the titers for Day 29 showed a dose-dependent increase, with no NAB detected in Group 1.

### Analysis of rAAV2hAQP1 vector DNA Biodistribution

**Figure 6:**
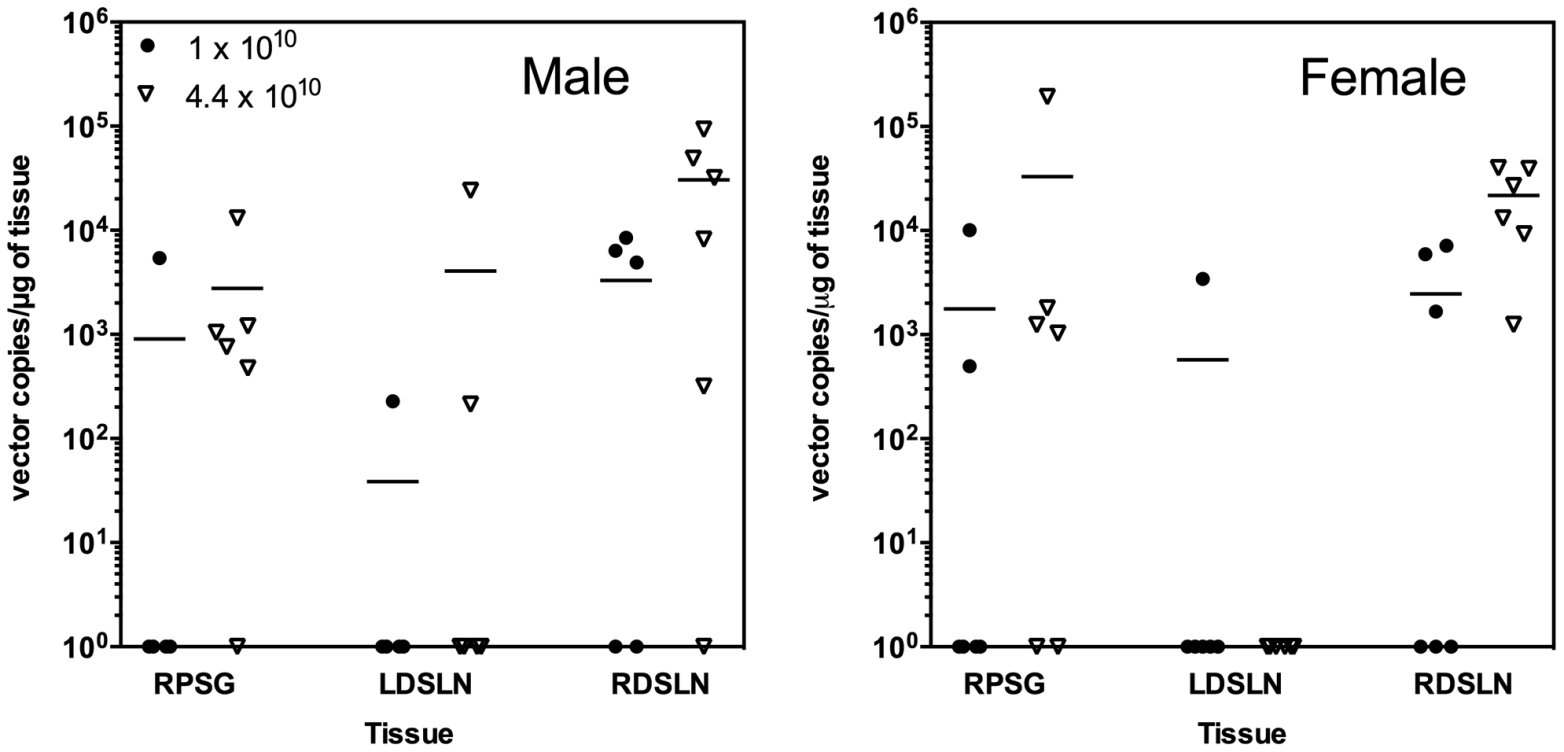
Distribution of rAAV2hAQP1 DNA detection on day 92 in tissue with > 50 copies/μg tissue for animals treated with the highest two doses, 1×10^10^ and 4.4×10^10^ (males, n = 6; females, n = 6). Vector distribution spiked on day 3, and was detected in blood and some saliva, but was cleared to levels below 50 copies/μg by the end of the 13 wk period except in RPSG, Right Parotid Salivary Gland; LDSLN, Left Draining Submandibular Lymph Node; and RDSLN, Right Draining Submandibular Lymph Node.

A total of 15 different organs were collected from each mouse and tested for vector DNA. By day 92 all had low copy number (< 50 copies/μg tissue) except for the salivary glands and draining lymph nodes. No vector was present in vehicle-treated group 1 and very low levels were present in groups 2 and 3 on any of the days, except for the parotid gland and draining lymph nodes. Data for rAAV2hAQP1 distribution in mouse tissue on day 92 was > 50 copies/ μg tissue for males and females as shown in Figure 6. Vector was detected in groups 4 and 5, which were treated with the highest vector doses, in the draining submandibular lymph node of some animals as well as the target gland (right parotid). No vector (> 50 copies/μg tissue) was found in the left submandibular gland for any of the animals from the four treatment groups. Vector genomes were found in the right and left parotid salivary gland. There were no significant gender or vector concentration differences for vector distribution for any of the sampled tissues. Vector was detected in blood in a dose-dependent manner only on day 3, with no detectable levels on later days. No vector DNA was detected in saliva past day 3. No animals had detectable vector DNA levels in the brain, duodenum, liver, lungs, or reproductive tissue past day 57.

## Discussion

Animals appeared to thrive well with no changes in body weight induced by the vector. This is in contrast to the Zheng *et al*. 2006 [7] 92-day AdhAQP1 toxicology study in rats, where a dose-related decrease in mean body weight was observed in females, but not in males. Food and water consumption remained unchanged between all the groups for males and females in both studies.

In contrast to vector studies in the submandibular gland that reported a gender related differences in vector copy number, no gender related difference was noted in the parotid glands [8]. Limited gender differences were seen in a higher NAB levels for females treated with lower doses of vector, 10^8^ and 10^9^ vp/gland, compared to males. Clinical hematology differences between genders were limited to the highest dose, 4.4×10^10^ vp/gland for WBC and SEGS levels on day 57, and control group HCT (day 92) and SEGS (day 3), suggesting no correlation with the treatment, and thus, biologically irrelevant.

Hematology evaluation also showed elevated WBC, SEGS, and lymphocyte levels for one male and two females cannulated with 4.4×10^10^ vp/gland compared to the rest of the animals in the cohort that did not have detectable vector DNA in the target tissue by day 92. Because vector DNA was detected in the right submandibular lymph node of all three animals, it is believed that the vector was delivered outside of the target tissue due to the small size of the mouse salivary glands. This should not be a concern with human application because of the much larger size and easier access to the human parotid gland. Unlike with serotype 5 adenoviral vector delivery [7], where dose and vector dependent inflammatory increase in rats was noted, none of the microscopic findings with AAV2 were considered to be test article induced. Mild to moderate inflammation in the right and left parotid glands was seen across all groups, with more animals experiencing left parotid gland inflammation (6 males and 7 females on day 3) than inflammation of the injected right parotid gland (3 males and 6 females) on day 3. Because by day 92, only one animal had inflammation limited to right parotid gland, this inflammation was determined to be surgically induced. In addition, none of the lesions observed were considered to be due to vector. This further suggests safe clinical applications of AAV2 vector delivery to the parotid gland.

Aquaporin channels, especially AQP1, are promising targets in restoring salivary gland hypofunction [9], [10]. A Phase 1 clinical trial was recently completed where AQP1 encoding adenoviral vector was used to restore salivary flow in eleven patients, using an open label, single dose, dose-escalation design [3]. After receiving a vector injection into a previously irradiated parotid gland, all patients tolerated the procedures well. Five of eleven patients showed positive response, all exposed to <5.8×10^9^ vp/gland. Higher vector doses did not yield positive responses likely as a result of an immune response to the adenovirus vector. To avoid this immune response, we have explored the possibility of using AAV vectors, which are less immunogenic and reported to have longer persistence in slowly dividing or post-mitotic tissue.

Adeno-associated viral gene transfer was initially considered unspecific and some toxicity was reported in transfected cells [11]. But much advancement in virology and vector preparation has been made and current studies with AAV vectors have reported limited immune response and toxicity. Vector tissue distribution analysis in our study demonstrated that vector remained localized to the target gland and left and right submandibular lymph nodes at the end of the study. The treatment was well tolerated, with no adverse clinical pathology effects. The NAB titer increased with respect to time and dose, however this was expected based on previous studies. In summary, no vector related toxicity was observed over the course of this study suggesting the treatment is clinically safe, and would be appropriate for advancement to a Phase 1 clinical trial.
